# Intergenerational Transmission of Paternal Epigenetic Marks: Mechanisms Influencing Susceptibility to Post-Concussion Symptomology in a Rodent Model

**DOI:** 10.1038/s41598-017-07784-7

**Published:** 2017-08-02

**Authors:** Harleen Hehar, Irene Ma, Richelle Mychasiuk

**Affiliations:** Alberta Children’s Hospital Research Institute, Hotchkiss Brain Institute, University of Calgary, Department of Psychology, Calgary, Alberta, Canada

## Abstract

Epigenetic transmission of phenotypic variance has been linked to paternal experiences prior to conception and during perinatal development. Previous reports indicate that paternal experiences increase phenotypic heterogeneity and may contribute to offspring susceptibility to post-concussive symptomology. This study sought to determine if epigenetic tags, specifically DNA methylation of promoter regions, are transmitted from rodent fathers to their sons. Using MethyLight, promoter methylation of specific genes involved in recovery from concussion and brain plasticity were analyzed in sperm and brain tissue. Promoter methylation in sperm differed based on paternal experience. Differences in methylation were often identified in both the sperm and brain tissue obtained from their sons, demonstrating transmission of epigenetic tags. For certain genes, methylation in the sperm was altered following a concussion suggesting that a history of brain injury may influence paternal transmission of traits. As telomere length is paternally inherited and linked to neurological health, this study examined paternally derived differences in telomere length, in both sperm and brain. Telomere length was consistent between fathers and their sons, and between brain and sperm, with the exception of the older fathers. Older fathers exhibited increased sperm telomere length, which was not evident in sperm or brain of their sons.

## Introduction

It is not surprising that maternal contributions to offspring development and disease susceptibility have been grossly studied^1–10^, as the mother is responsible for influencing development throughout pregnancy and the maternal egg represents a high investment in the offspring. Conversely, for decades, the sperm has been regarded as a passive donor of genetic information, with a limited role in altering developmental trajectories of offspring^11^. However, emerging insights into epigenetic mechanisms and paternal inheritance have questioned this assumption, suggesting that sperm can carry information that has potential to drive changes in offspring gene expression and consequently affect phenotype^12^. In addition to genetic changes to the DNA sequence itself, histone modifications, methylation changes, as well as the type and quantity of microRNAs present in the sperm can affect various mechanisms in a developing organism, thus contributing to long-term changes in molecular pathways, behaviour, and susceptibility to disease^13^.

Consequently, there have been many groundbreaking discoveries that demonstrate paternal experiences can alter behaviour and disease susceptibility of offspring^12, 14–20^. When examining disease susceptibility, certain paternal characteristics have been associated with specific conditions in offspring. For example, advancing paternal age (AA) has been associated with increased risk of various neurological disorders such as schizophrenia and autism whereas paternal high fat diet (HFD) has been associated with increased mortality in male offspring^18, 20–23^. A recent study in our laboratory demonstrated that paternal experiences prior to mating, specifically HFD consumption and AA, were associated with altered behaviour, gene expression, and varied post-concussive symptom presentation^24^. However, a fundamental question that remains unanswered is what drives these changes in offspring behavioural and molecular profiles; in other words, how is paternal experience transmitted to subsequent generations?

Given that DNA methylation changes are firmly believed to play a role in epigenetic inheritance, regulating gene expression, and subsequently affecting physiological processes and behaviour^25–27^, we hypothesized that DNA methylation was a significant modulator of intergenerational transmission of paternal experience. DNA methylation is the primary epigenetic contributor to the stable maintenance of a gene’s given expression state^28^ and is generally associated with gene silencing^29^. Unlike oogenesis, spermatogenesis is an ongoing process that begins at puberty and continues throughout a male’s lifespan; DNA methylation is also maintained during this process and modifications to methyl tags occur throughout spermatozoa development^12^. As environmental factors are able to epigenetically modify DNA methylation in developing sperm, paternal experiences provide a conduit for phenotypic variation in their offspring.

Traumatic brain injury is one of the leading causes of death and disability in North America, with mild traumatic brain injury or concussion (mTBI) comprising the greatest proportion of this endemic^30^. For children and adolescents, concussion is most often associated with sports related injuries, falls, and automobile accidents^31^. While many recover rapidly (within 7–10 days) and without overt manifestation of injury pathology, a significant proportion suffers from a persistent complement of symptoms termed post-concussive syndrome (PCS)^32^. One of the most puzzling aspects of PCS is that it does not affect everyone equally. This ambiguity in symptom presentation and prognosis is likely a reflection of the complexity of mTBI pathophysiology and the heterogeneity of premorbid characteristics^33^. Pre-existing differences, in neuronal and metabolic functioning, such as paternal epigenetic transmission, may render some individuals susceptible to PCS and others resilient.

The purpose of this study was therefore twofold. The first purpose was to examine the epigenetic regulation of gene expression, where the first aim was to determine if AA and HFD paternal experiences do in fact alter DNA methylation in developing sperm. The second aim was designed to investigate the transmission of DNA methylation from fathers to their offspring. Thus, we compared methylation patterns in sperm of male offspring and fathers within each treatment group. The third aspect of the study sought to determine whether or not DNA methylation in sperm was representative of DNA methylation within the brain. And finally, the study investigated the effect of mTBI on DNA methylation in developing offspring sperm. The second purpose of this study was to examine the paternal transmission of telomere length (TL) by investigating its epigenetic regulation. Recent evidence suggests that telomeres, repetitive non-coding sequences of DNA on ends of eukaryotic chromosomes, play an active role in epigenetic patterning, with shorter telomere length (TL) often being associated with progression of neurodegenerative diseases^34–38^. Previous research has demonstrated that telomeres are responsive to many environmental manipulations such as exercise^39–41^ and a previous study in our laboratory demonstrated that TL can be used as a prognostic biomarker of concussion^42^.

To do this, DNA methylation in the promoter region of 6 genes believed to play a significant role in neurodevelopment, injury response, and the mediation of advancing age and high fat diet consumption (*Bdnf, Lept-R, Oxy-R*, *Tert, Igf2, and Igf2-R*) was examined. Although this is not an exhaustive exploration of epigenetic change, this study examined the transmission of DNA methylation in the promoter regions of 6 specific genes from fathers to their offspring. Brain derived neurotrophic factor (*Bdnf*) has been demonstrated to promote brain plasticity and regulate neuronal development and survival^43^. In addition, the neuroprotective properties of *Bdnf* have been exhibited in TBI^44, 45^, but loss of this neurotrophic factor has also been implicated in neurodevelopmental disorders such as ADHD^46^. Leptin’s primary role is signaling satiety and reducing an individual’s motivation to consume food^47^. Additionally, leptin reduces stress hormone levels and promotes brain plasticity and development^48, 49^. The leptin receptor (*Lept-R*) binds leptin and in turn regulates leptin-dependent processes. Oxytocin is a hormone and neuropeptide that has been implicated in the promotion of social behaviour, attachment, and pair bonding, by reducing anxiety^50, 51^. Additionally, oxytocin plays a significant role in paternal-offspring interactions and receptor binding (*Oxy-R*) for oxytocin appears to be transmitted from fathers to their sons^52^. *Tert* is a component of telomerase, a reverse transcriptase involved in the elongation of telomeres, has anti-apoptotic functions, and has been implicated in the prevention of aging-dependent neurodegeneration^53–56^. Finally, insulin-like growth factor 2 (*Igf2*) is a polypeptide hormone with neurotrophic activity that has been associated with brain development^57^. Important to this study, *Igf2* is a paternally imprinted gene, indicating that the allele from the father is methylated in germline cells and in-turn silenced. Conversely, the insulin-like growth factor receptor (*Ifg2-R*) is maternally imprinted, indicating that the allele from the mother is methylated in germline cells and in-turn silenced. This gene functions to clear *Igf2* from the cell surface and attenuate signaling processes^58^.

## Results

### Epigenetic Regulation of Gene Expression

#### Examination of Promoter Methylation in Father’s Sperm

The initial question under investigation was whether or not manipulating paternal age or diet would alter methylation patterns in the father’s sperm. Results from the one-way ANOVAs comparing methylation levels in AA, HFD, and Control sperm, demonstrated that paternal experience did alter methylation patterns of the gene promoter regions in 5/6 genes investigated. Post-hoc analyses were performed when appropriate and all results can be found in Table 1.

**Table 1:** Results from the one-way ANOVAs examining promoter methylation in sperm from fathers in the three treatment conditions.

Gene	Main Effect F(*p*)	Post-hoc Analyses		
		AA – Control	HFD – Control	AA - HFD
*Bdnf*	513.89 (<0.01)	<0.01	<0.01	<0.01
*Lept-R*	6.34 (0.02)	0.05	0.22	<0.01
*Oxy-R*	31.46 (<0.01)	0.02	<0.01	<0.01
*Tert*	6.52 (0.02)	0.13	0.05	<0.01
*Igf2*	2.52 (0.15)	N/A	N/A	N/A
*Igf2-R*	6.11 (0.03)	0.92	0.04	0.04

#### Comparison of Promoter Methylation in Sperm Between Fathers and Sons

The second question was whether or not methylation levels in the promoter regions for the specific genes was similar for fathers and their sons. In other words, is the methylation pattern for a specific gene transmitted from the father’s sperm to the sperm of his sons? One-way ANOVAs with Generation (Father, Son) as factors were conducted for the sperm methylation levels in each treatment condition for all of the genes. Father and son methylation patterns did not differ for the *Bdnf, Lept-R*, *Tert*, and *Igf2* in the AA group, the *Bdnf, Oxy-R, Tert, Igf2*, and *Igf2-R* in the HFD group, and the *Bdnf*, *Tert, Igf2*, and *Igf2-R* in the Control group. See Table 2 for statistical results.

**Table 2:** Results from the one-way ANOVAs examining promoter methylation in the sperm obtained from fathers and their sons.

Gene	Main Effect F(*p*)		
	**AA**	**HFD**	**Control**
*Bdnf*	0.03 (0.87)	6.11 (0.06)	0.21 (0.67)
*Lept-R*	0.05 (0.83)	27.16 (<0.01)	8.97 (0.02)
*Oxy-R*	56.34 (<0.01)	4.50 (0.09)	23.67 (<0.01)
*Tert*	1.00 (0.36)	1.32 (0.30)	1.37 (0.29)
*Igf2*	2.23 (0.17)	0.00 (0.99)	0.59 (0.46)
*Igf2-R*	5.05 (0.05)	3.09 (0.11)	0.03 (0.86)

#### Comparison of Promoter Methylation in Sperm and Brain

The third question was whether or not the methylation for a given gene was similar in the sperm and brain. To examine the relationship between the level of methylation in the brain and sperm of the sons, one-way ANOVAs with Tissue Sampled (brain, sperm) as factors were conducted for each gene per specific Paternal Treatment group. The analysis demonstrated that for most genes in each of the treatment groups, promoter methylation was consistent in sperm and brain. However, the *Oxy-R* was differentially methylated in sperm and brain for all treatment groups, *Tert* exhibited increased methylation in sperm when compared to brain for AA and HFD sons, *Bdnf* was significantly more methylated in sperm when compared to brain in the control sons, and *Igf2* was differentially methylated in sperm and brain for AA and control sons. See Table 3 for all statistical results.

**Table 3:** Statistical results for the one-way ANOVAs that examined differences in promoter methylation in sperm and brain obtained from sons.

Gene	Main Effect F(*p*)		
	**AA**	**HFD**	**Control**
*Bdnf*	0.30 (0.60)	2.57 (0.16)	10.33 (0.02)
*Lept-R*	2.12 (0.20)	0.16 (0.70)	3.81 (0.10)
*Oxy-R*	21.11 (<0.01)	68.17 (<0.01)	124.64 (<0.01)
*Tert*	0.04 (0.84)	6.38 (0.05)	3.10 (0.13)
*Igf2*	6.19 (0.03)	2.57 (0.16)	22.25 (<0.01)
*Igf2-R*	0.06 (0.81)	1.94 (0.18)	0.03 (0.86)

#### Examination of the Effects of mTBI on Promoter Methylation in Sperm of Sons

In addition to investigating paternal transmission of promoter methylation status from sperm of fathers to their sons, the influence of mTBI on sperm methylation patterns was also determined. To study if the mTBI at P30 altered methylation of these 6 promoter regions, separate one-way ANOVAs with Injury (mTBI, Sham) as factors were performed for each of the genes for each of the Paternal Treatment groups. The mTBI altered methylation of the *Oxy-R* in the sperm of sons born to HFD fathers, and in *Tert* of sperm from sons of AA and Control fathers, and *Bdnf* in sons born to HFD and Control fathers. See Table 4 for statistical results.

**Table 4:** Results from the ANOVAs used to examine promoter methylation in the sperm of sham and mTBI sons from the three paternal treatment groups.

Gene	Main Effect F(*p*)		
	**AA**	**HFD**	**Control**
*Bdnf*	0.02 (0.89)	33.77 (<0.01)	14.29 (0.01)
*Lept-R*	1.94 (0.21)	0.50 (0.51)	0.04 (0.85)
*Oxy-R*	2.05 (0.20)	7.26 (0.03)	0.34 (0.58)
*Tert*	5.87 (0.05)	0.06 (0.82)	5.63 (0.05)
*Igf2*	1.56 (0.26)	2.04 (0.11)	2.69 (0.14)
*Igf2-R*	3.88 (0.08)	1.98 (0.21)	0.72 (0.43)

**Figure 1:**
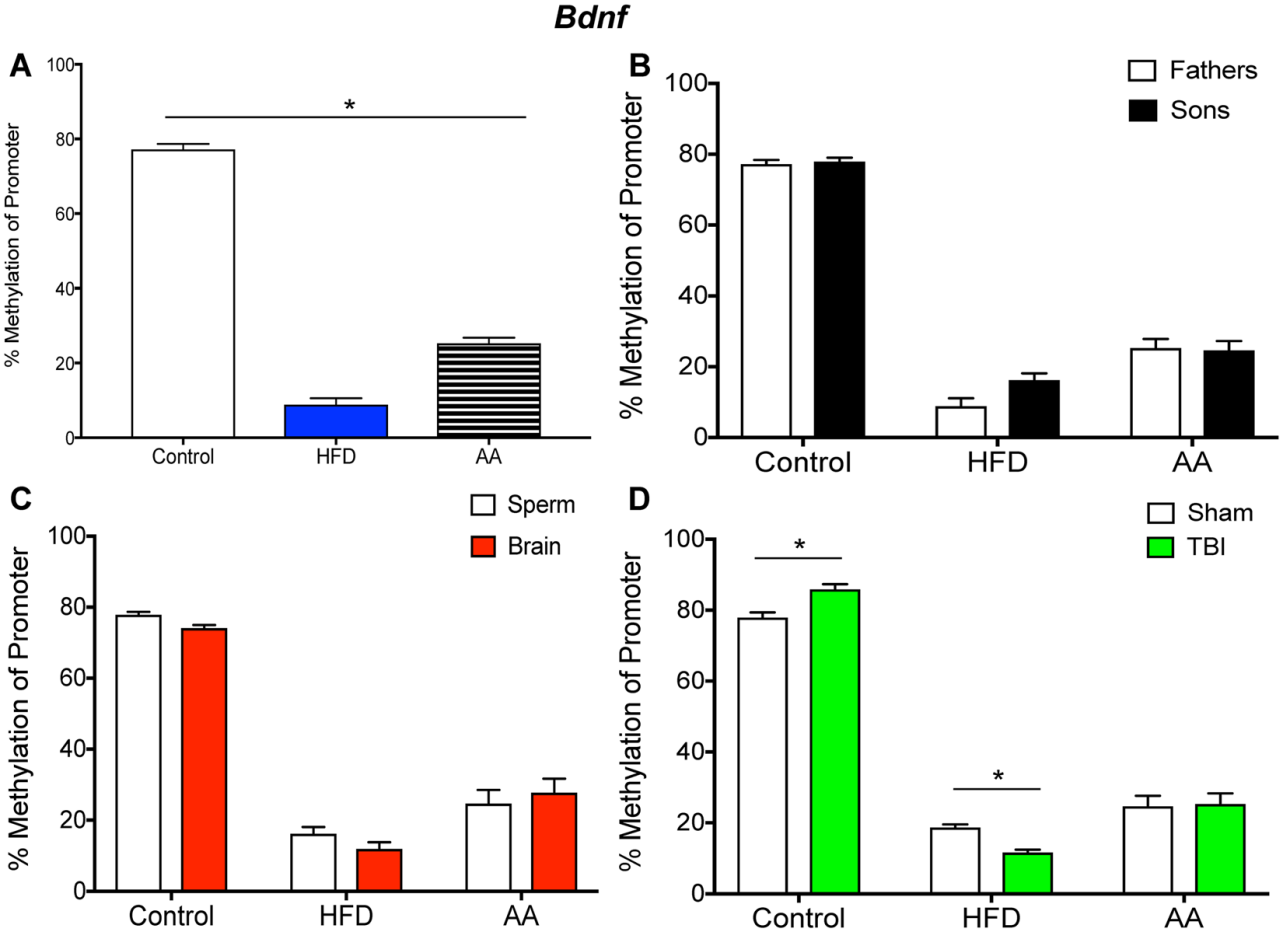
Illustrative demonstrations of *Bdnf* promoter methylation. One way ANOVAs were conducted and significant main effects are indicated (*p < 0.05). (**A**) Average *Bdnf* promoter methylation in paternal sperm was significantly reduced in HFD and AA sperm compared to control sperm (*p* < 0.05). (**B**) There were no significant differences in *Bdnf* promoter methylation between father and son sperm in either of the paternal treatment conditions (*p* > 0.05). (**C**) There were no significant differences in *Bdnf* promoter methylation between sperm and brain of offspring (*p* > 0.05). (**D**) mTBI in control offspring was associated with an increased *Bdnf* methylation whereas mTBI in HFD offspring was associated with decreased *Bdnf* promoter methylation (*p* < 0.05).

**Figure 2:**
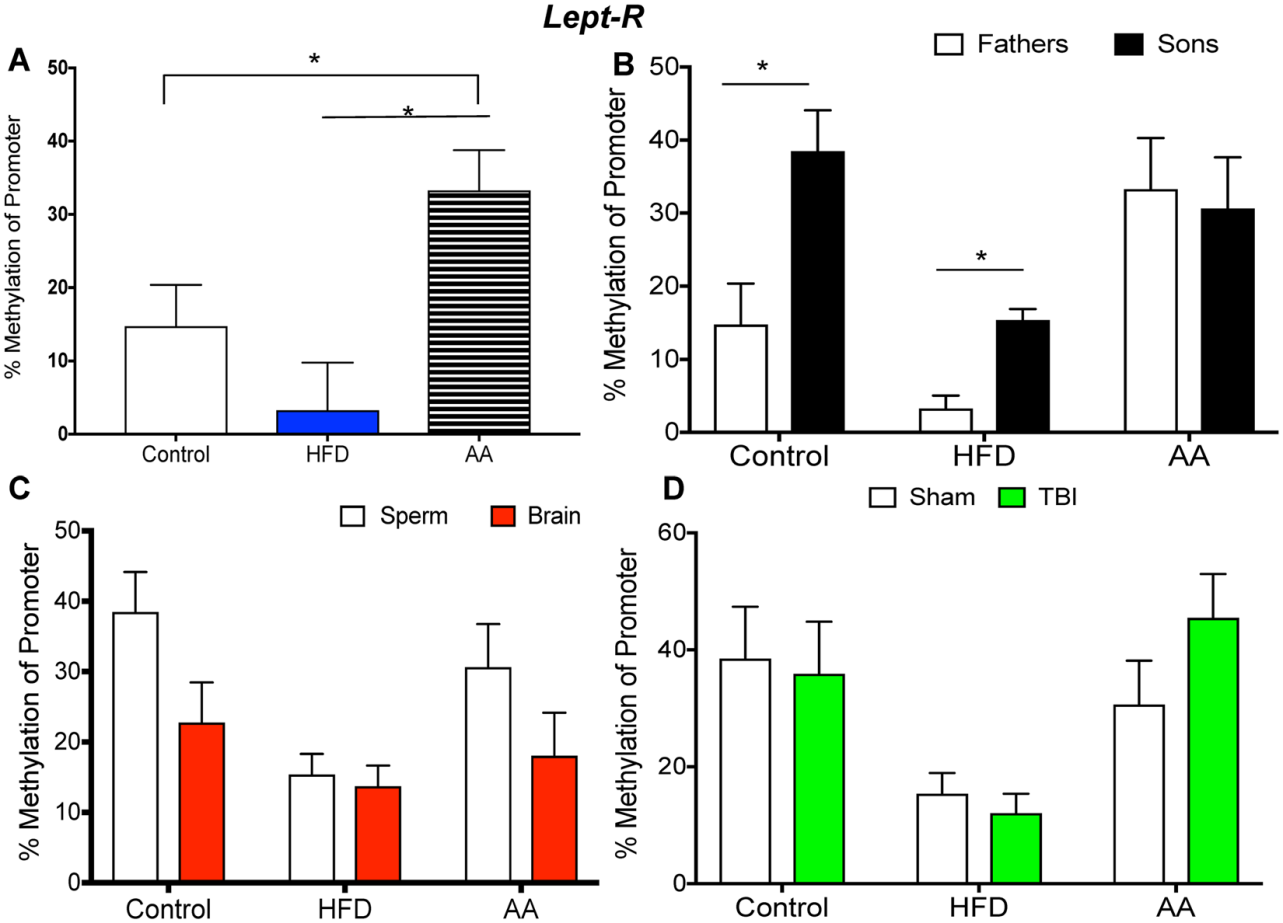
Illustrative demonstrations of *Lept-R* promoter methylation. One way ANOVAs were conducted and significant main effects are indicated (*p < 0.05). (**A**) Average *Lept-R* promoter methylation in paternal sperm was significantly increased in AA sperm compared to control and HFD sperm (*p* < 0.05). (**B**) *Lept-R* promoter methylation was significantly increased in sperm of sons of control and HFD fathers compared to paternal sperm (*p* < 0.05). (**C**) There were no significant differences in *Lept-R* promoter methylation between sperm and brain of offspring (*p* > 0.05). (**D**) There were no significant differences in *Lept-R* promoter methylation between sham and mTBI groups (*p* > 0.05).

**Figure 3:**
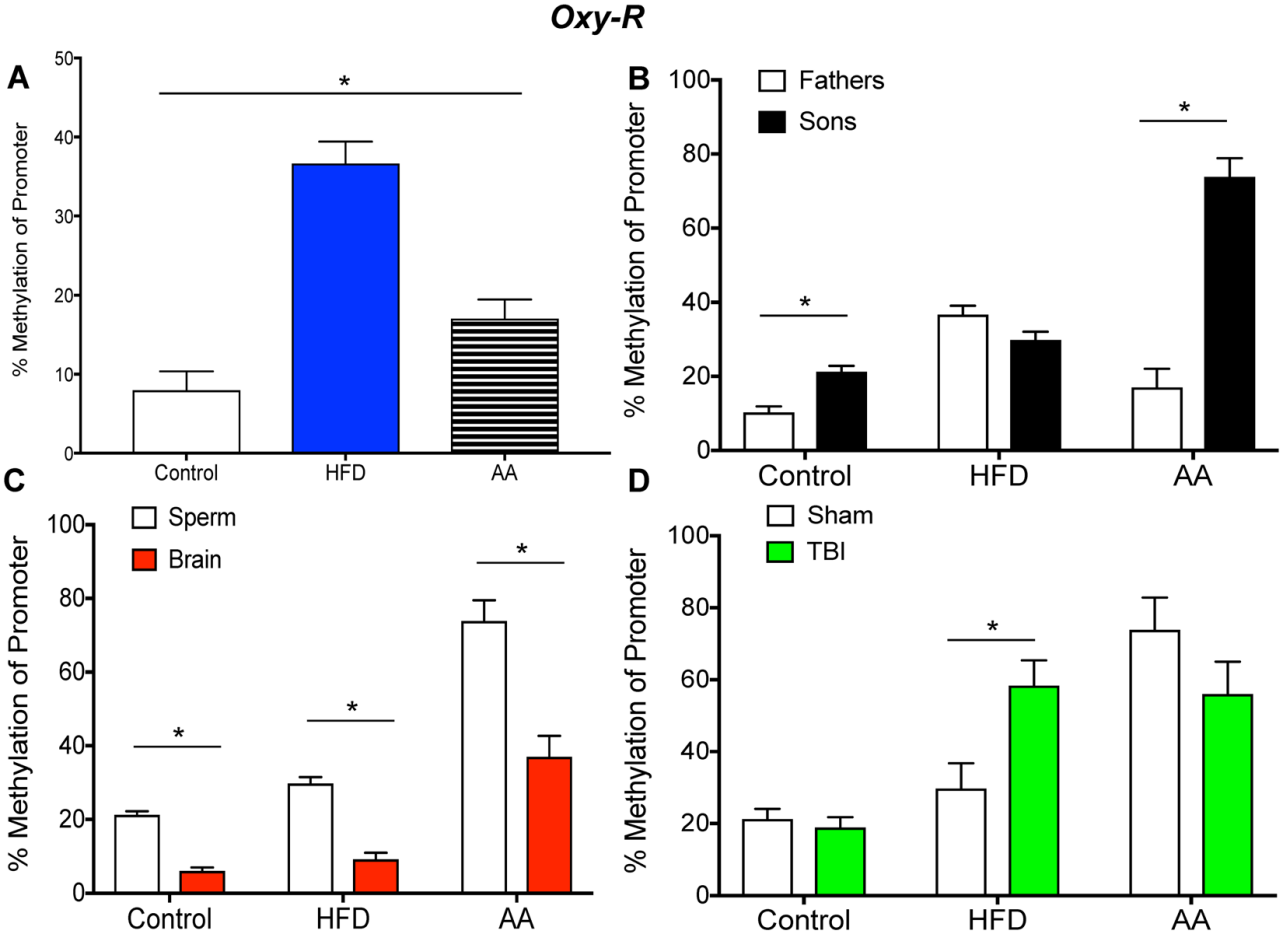
Illustrative demonstrations of *Oxy-R* promoter methylation. One way ANOVAs were conducted and significant main effects are indicated (*p < 0.05). (**A**) Average *Oxy-R* promoter methylation in paternal sperm was significantly increased in HFD sperm compared to control and AA sperm (*p* < 0.05). (**B**) *Oxy-R* promoter methylation was significantly increased in sperm of sons of control and AA fathers compared to paternal sperm (*p* < 0.05). (**C**) *Oxy-R* methylation was significantly reduced in offspring brain compared to sperm in all three treatment groups (*p* < 0.05). (**D**) *Oxy-R* methylation was significantly increased in mTBI sons compared to sham sons of HFD fathers (*p* < 0.05).

**Figure 4:**
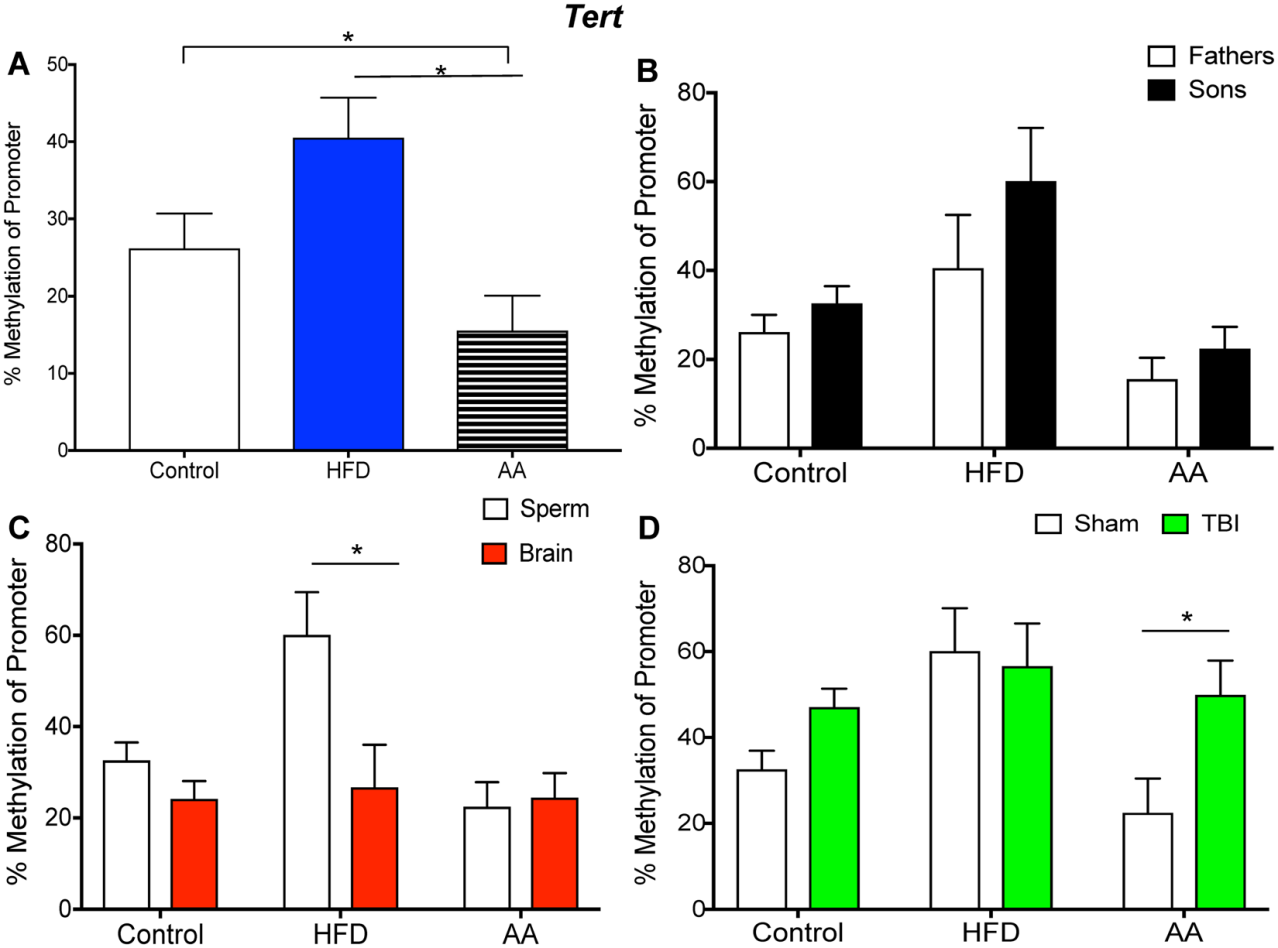
Illustrative demonstrations of *Tert* promoter methylation. One way ANOVAs were conducted and significant main effects are indicated (*p < 0.05). (**A**) Average *Tert* promoter methylation in paternal spermwas significantly increased in HFD sperm compared to AA and control sperm (*p* < 0.05). (**B**) There were no significant differences in *Tert* promoter methylation between father and son sperm in all three paternal treatment conditions (*p* > 0.05). (**C**) *Tert* promoter methylation in the sperm was significantly higher than brain methylation in HFD offspring (*p* < 0.05). (**D**) The mTBI in AA offspring was associated with an increased *Tert* methylation (*p* < 0.05).

**Figure 5:**
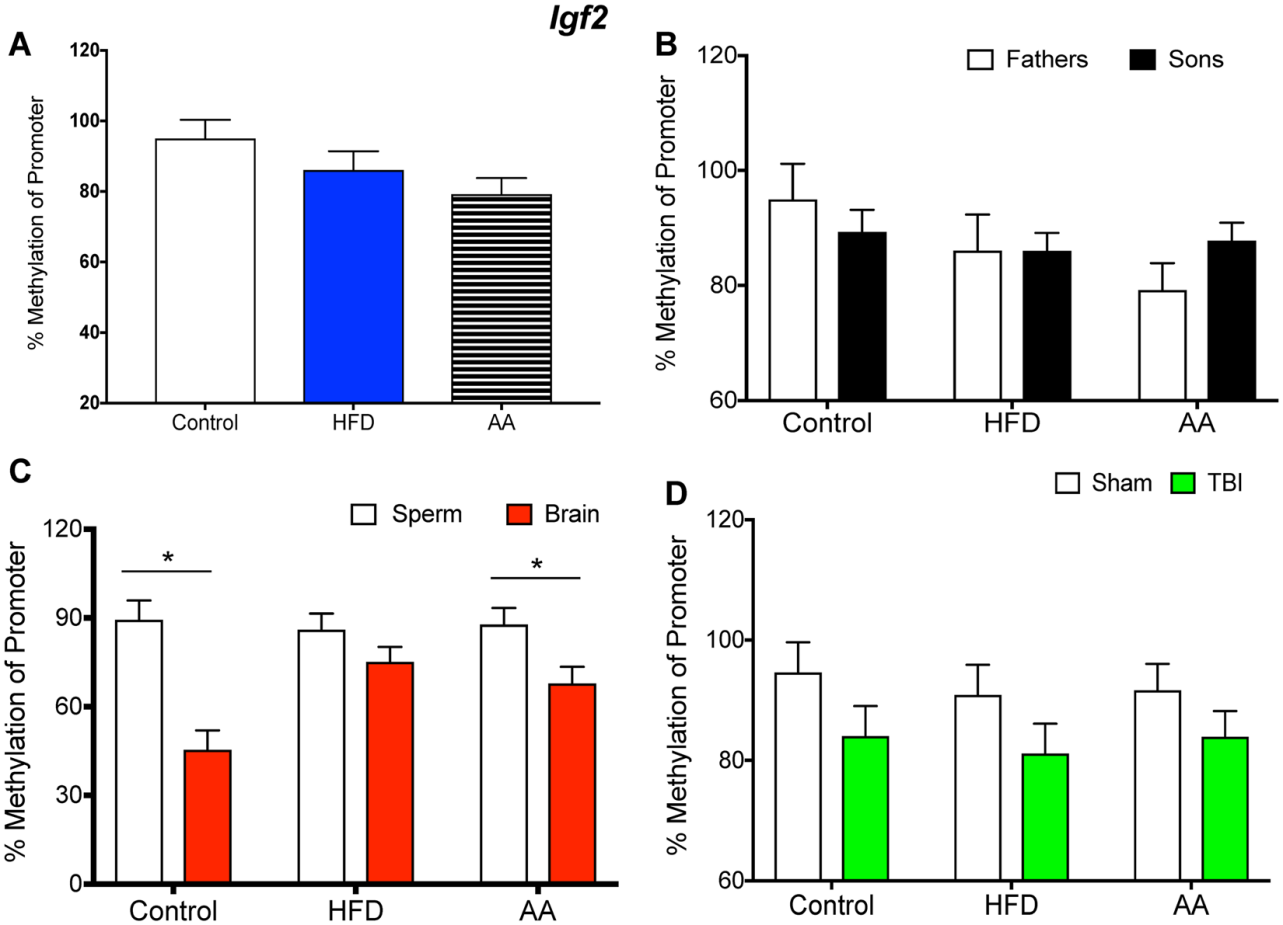
Illustrative demonstrations of *Igf2* promoter methylation. One way ANOVAs were conducted and significant main effects are indicated (*p < 0.05). (**A**) Average *Igf2* promoter methylation in paternal sperm was not affected by paternal experience. (**B**) There were no significant differences in *Igf2* promoter methylation between father and son sperm in all three paternal treatment conditions (*p* > 0.05). (**C**) *Igf2* promoter methylation in the sperm was significantly higher than brain methylation in Control and AA offspring (*p* < 0.05). (**D**) The mTBI did not affect methylation of the *Igf2* promoter (*p* > 0.05).

**Figure 6:**
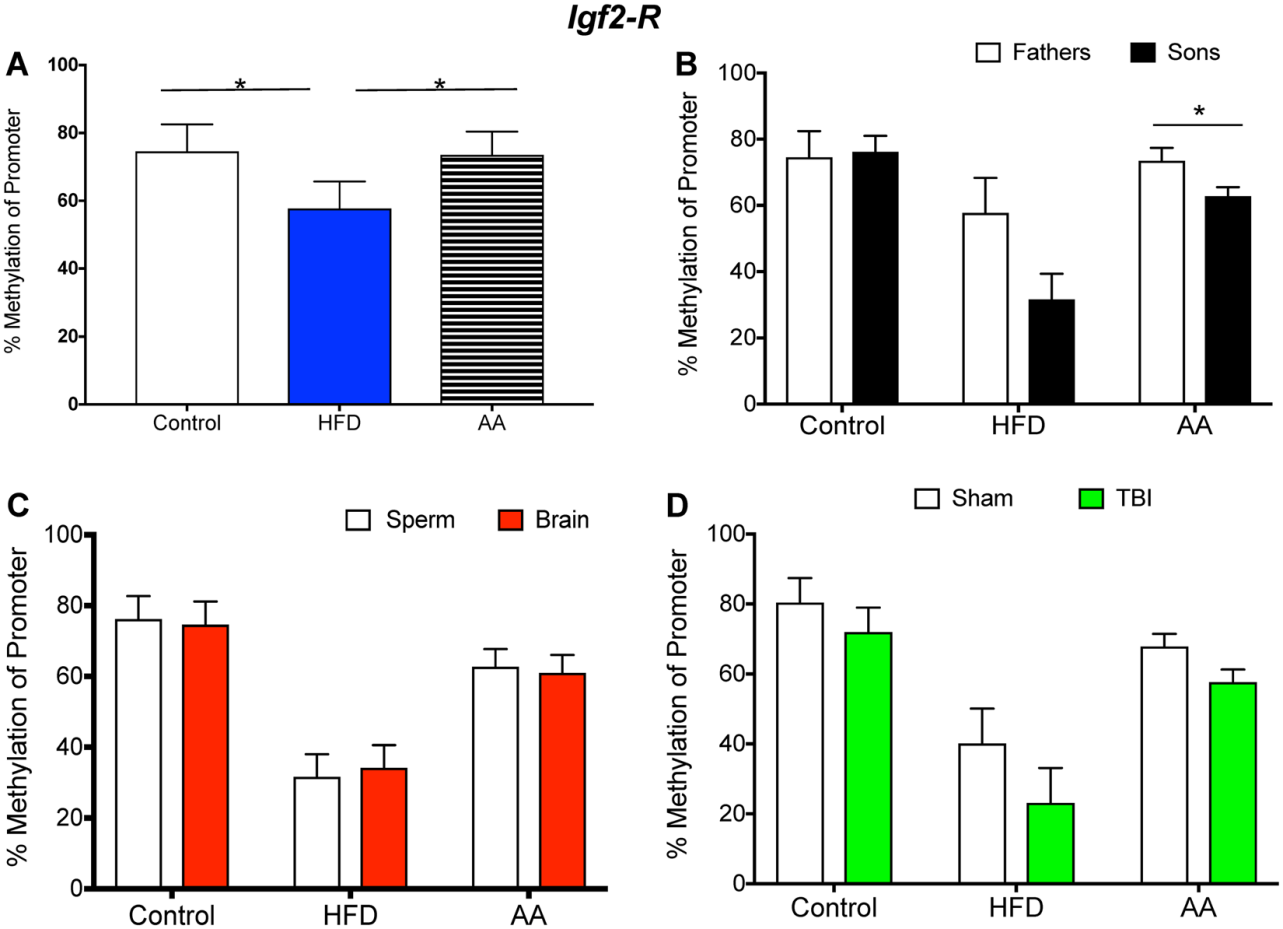
Illustrative demonstrations of *Igf2-R* promoter methylation. One way ANOVAs were conducted and significant main effects are indicated (*p < 0.05). (**A**) Average *Igf2-R* promoter methylation in paternal sperm was significantly decreased in HFD sperm compared to AA and control sperm (*p* < 0.05). (**B**) Significant differences in *Igf2-R* promoter methylation were identified between father and son sperm in the AA paternal treatment condition (*p* < 0.05). (**C**) *Igf2-R* promoter methylation in the sperm did not differ from promoter methylation in the brain for any of the conditions (*p* > 0.05). (**D**) Similarly, the mTBI did not alter promoter methylation of the *Igf2-R* in control, HFD or AA offspring (*p* > 0.05).

Figures 1–6 illustrate promoter methylation for the specific genes of interest (Fig. 1. *Bdnf*; Fig. 2. *Lept-R*; Fig. 3. *Oxy-R*; Fig. 4. *Tert*; Fig. 5, *Igf2*; Fig. 6, *Igf2-R*) as they pertain to the four aspects of investigation outlined above.

#### *Examining the Relationship Between Methylation Level and Gene Expression in Brain*

Given that increased methylation is generally believed to be associated with decreased gene expression, methylation level and gene expression in the hippocampus (HPC) were compared for each of the specific genes. Pearson’s correlations between methylation level and expression were determined for each of the genes for the given paternal treatment groups. For sons in the control group, a significant negative correlation was identified for methylation and gene expression for *Lept-R* and *Tert*. Sons in the HFD group also exhibited a significant negative correlation between methylation and gene expression for the *Lept-R* and the *Igf2-R*. Sons in the AA group showed a significant negative correlation for the methylation and gene expression for *Igf2* and *Igf2-R*. Although no other significant correlations were identified, all groups exhibited a negative relationship demonstrating that overall, increased methylation was associated with a decrease in expression. See Table 5 for statistical findings.

**Table 5:** Results from the correlational analysis carried out between promoter methylation and expression level for each of the genes of interest.

Gene	Pearson’s Correlation r (*p*)		
	**AA**	**HFD**	**Control**
*Bdnf*	−0.41 (0.09)	−0.51 (0.07)	−0.40 (0.10)
*Lept-R*	−0.03 (0.48)	−0.60 (0.05)	−0.68 (0.03)
*Oxy-R*	−0.42 (0.15)	−0.05 (0.46)	−0.42 (0.12)
*Tert*	−0.10 (0.40)	−0.43 (0.14)	−0.62 (0.05)
*Igf2*	−0.63 (0.01)	−0.19 (0.35)	−0.22 (0.31)
*Igf2-R*	−0.53 (0.01)	−0.47 (0.02)	−0.17 (0.44)

### Epigenetic Regulation of Telomere Length

#### Do Paternal Experiences Modify Telomere Length in Sperm

**Figure 7:**
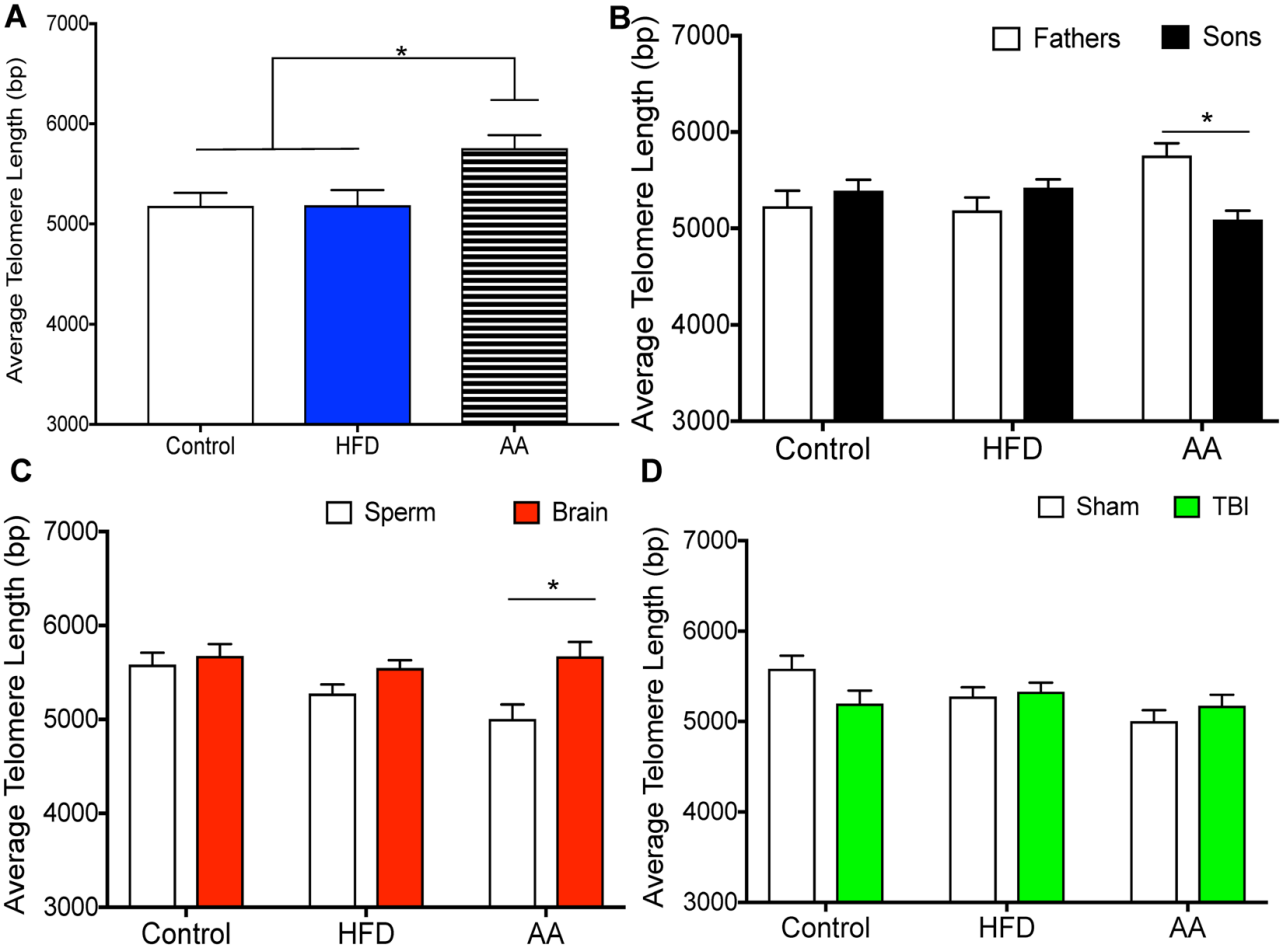
Illustrative example of average telomere length (TL). One way ANOVAs were conducted and significant main effects are indicated (*p < 0.05). (**A**) Average TL in paternal sperm samples was significantly longer in AA fathers compared to control and HFD groups (*p* < 0.05). (**B**) Sperm TL was significantly shorter in AA offspring compared to fathers (*p* < 0.05). There were no significant differences in TL between father and son sperm in the other two paternal treatment conditions (*p* > 0.05). (**C**) TL was significantly longer in the brain of AA offspring compared to offspring sperm (*p* < 0.05). (**D**) There were no significant differences in TL between sham and mTBI groups (*p* > 0.05).

In addition to changes in gene expression, the study also sought to determine if paternal experience altered telomere length, and whether or not these changes could be transmitted to their sons. See Fig. 7A. First, a one-way ANOVA with Paternal Treatment as the factor was conducted on telomere length in paternal sperm samples. Telomere length in sperm from AA fathers was significantly longer than that of controls or HFD. The one-way ANOVA exhibited a significant main effect of paternal treatment *F*(2, 10) = 6.07, *p* = 0.02, with post-hoc analyses demonstrating significant differences between AA – Controls (*p* = 0.01), and AA – HFD (*p* = 0.02), but not between HFD – Controls (*p* = 0.97).

#### Examination of Telomere Length in Sperm from Father’s and their Sons

Similar to the gene expression analysis, the second question was, if paternal experience modifies telomere length in sperm, are the changes also present in the sperm of their sons? Three, one-way ANOVAs with Generation (Fathers, Sons) as the factor were performed for telomere length obtained from the sperm of dads and their offspring. Telomere length was significantly different in fathers and sons from the AA group, *F*(1, 12) = 16.34, *p* < 0.01, but not in the HFD or control group, *p*’s > 0.05 (see Fig. 7B).

#### Examination of Telomere Length in the Sperm and Brain of Sons

The study sought to determine if the average telomere length obtained in sperm samples was similar to that of the average telomere length in the brain. One-way ANOVAs with Tissue (Sperm, Brain) as factors were run for telomere length in each of the paternal treatment conditions. Significant differences in TL were identified between sperm and brain of AA sons (*F*(1, 15) = 9.27, *p* = 0.02), but not between sperm and brain in HFD or Control sons, *p* = 0.09 and 0.62, respectively (see Fig. 7C).

#### *Investigating the Effects of mTBI on Telomere Length in Son’s Sperm*

Finally, the study determined if the mTBI altered telomere length in the sperm of the sons as this could have implications for future generations (Fig. 7D). Three one-way ANOVAs with Injury (mTBI, Sham) as the factor were conducted for telomere length in sperm obtained from the sons. The one-way ANOVAs failed to demonstrate significant differences for sperm telomere length between mTBI and sham animals in any of the three paternal treatment groups, (AA; *F*(1, 11) = 0.98, *p =* 0.36, HFD; *F*(1, 11) = 2.68, *p* = 0.16, Control; *F*(1, 11) = 3.43, *p* = 0.13).

#### Is Telomere Length Correlated with Tert Expression

Given that telomerase is responsible for the addition of telomeric repeats to the ends of chromosomes, we sought to determine if *Tert* expression was correlated with TL. TL and *Tert* expression levels from all sons were used. Pearson’s correlations between telomere length and *Tert* gene expression demonstrated a trend toward significance, *r* = 0.36, *p* = 0.08, indicating that *Tert* expression and TL were positively correlated, albeit not significantly.

## Discussion

This study sought to determine how paternal experiences could contribute to the large degree of heterogeneity that exists in symptom presentation and individual risk for PCS. Previous work demonstrated that AA and HFD in fathers prior to conception increased variability in offspring PCS presentation, with both paternal experiences modifying outcomes^24^. In an effort to understand the mechanisms by which paternal experiences could influence these outcomes, this study examined intergenerational transmission of DNA methylation in six specific genes (*Bdnf, Lept-R, Oxy-R, Tert, Igf2*, and *Igf2-R*). In the sperm from fathers, promoter methylation for 5/6 genes differed as a result of paternal experience. In many cases, promoter methylation did not differ in sperm from fathers and their sons, with sons also showing similar promoter methylation in their brain tissue. For certain genes the mTBI altered promoter methylation in sperm, suggesting that mTBI history has the potential to induce individual variability in the next generation.

These findings are consistent with paternal transmission theories that have postulated that paternal effects represent a source of variation for offspring phenotype and fitness^11^. This is an important concept because it provides fathers with a mechanism by which they can transmit beneficial information about the environment to their offspring to allow for greater degree of adaptation. We found that promoter methylation, and subsequent expression of the 6 genes investigated in offspring, was largely determined by the experiential differences of the fathers prior to conception. *Bdnf* exhibited the most pronounced and consistent transmission of promoter methylation from fathers to offspring; exhibiting strong associations between DNA methylation in sperm from fathers and their sons, and demonstrating robust relationships between sperm and brain methylation that was specific to the paternal experience prior to conception. The implications associated with paternal experience influencing expressional regulation of this gene are significant, given the important role *Bdnf* plays in development, injury response, and neuroplasticity^43, 45^.

Conversely, DNA methylation in the promoter of the oxytocin receptor, *Oxy-R*, was not consistently inherited from fathers to sons, nor was promoter methylation of this gene consistent in offspring sperm and brain. Of importance however, differences in methylation of the promoter were identified across paternal groups, and these differences were maintained in offspring sperm and brain. *Oxy-R* activity has been shown to be highly dependent upon the early environment, with rearing style (high licking and grooming dams vs. low licking and grooming dams), delayed weaning, and early handling, all influencing activity of the receptor^52, 59–61^. It is therefore possible that early environmental conditions modified methylation of the *Oxy-*R, preventing identification of father-to-son transmission of this gene expression state. Interestingly, even early life experiences were not strong enough to eliminate the effects of paternal experience on DNA methylation, as group differences were still evident in sperm and brain of offspring.

In addition, growing literature demonstrates that HFD consumption and obesity in fathers is associated with changes to the metabolic phenotype of their offspring via epigenetic programming^62–65^. Leptin and the leptin receptor are important regulators of food intake and body weight, but have also been implicated in the modulation of synaptic plasticity^66^. This study found that methylation of the promoter region in the *Lept-R* gene was altered by paternal experience, with HFD fathers exhibiting significant reductions in cytosine methylation, and AA fathers exhibiting significant increases in methylation. Although DNA methylation was consistent in sperm from AA fathers and their sons, sons in the HFD and Control groups exhibited significantly greater levels of DNA methylation than their fathers. The development of leptin resistant diet-induced obesity has been shown to occur in multiple stages and actually take months to manifest^67^. As fathers were only on the diet for 3 weeks prior to conception, the epigenetic transmission of HFD-associated factors is likely mediated through a different mechanism in our animals. Promoter methylation of the *Lept-R* gene did not differ between sperm and brain samples for the sons in any of the groups, nor was it affected by the mTBI.

The effect of paternal age has also become an emergent area of research as AA has been linked to many neurocognitive disorders including autism, schizophrenia, and bipolar disorder^14, 18–20, 23^. To examine epigenetic transmission from fathers to their offspring that may be associated with AA, this study investigated DNA methylation in the promoter of telomerase (*Tert*). *Tert* is a primary component of the telomerase enzyme complex that is responsible for maintaining telomeres and the integrity of cells, and in turn has been associated with aging processes^54, 68^. Similar to the other genes of interest, with the exception of *Igf2*, DNA methylation in the promoter region of *Tert* differed in the sperm of AA, HFD and Control fathers. The decreased methylation levels of *Tert* in sperm from AA fathers may represent increased *Tert* activity that may serve to compensate for telomere loss associated with increased cell turnover in older fathers. Methylation of the *Tert* promoter was consistent across sperm from fathers and their sons may be a contributor through compromising cell integrity. Promoter methylation was maintained between sperm and brain samples in the Control and AA sons, but differed for sons from the HFD group. As male obesity has been linked to reduced fertility and reproductive health^69^, the increased methylation of the *Tert* promoter in sperm of HFD fathers and their sons may be contributing to their sperm health and reduced reproductive success. Of importance, the mTBI did alter promoter methylation in sperm of sons born to AA fathers, increasing methylation, thereby reducing expression of *Tert*. Although this requires additional investigation, sons with a history of mTBI who were born to older fathers, may transmit additional risk to subsequent generations.

In addition, normal mammalian development is dependent upon a small number of imprinted genes that are specifically expressed from either the maternal or the paternal genome^70, 71^. Imprinting relies on a small group of genes that are epigenetically marked, often via DNA methylation by *de novo* DNA methyltransferases, to establish parent-of-origin-specific gene expression^71^. *Igf2* is a paternally imprinted gene and should therefore be silenced in male gametes. As expected, we found that the *Igf2* promoter region in sperm from both fathers and sons, across all treatment conditions, was between 85–95% methylated, suggesting gene silencing. Conversely, the *Ifg2-R* is maternally imprinted, silenced in female gametes, and therefore should be expressed in our male rats. In support of this, we found methylation of the promoter region for the *Igf2-R* to range from 30–75% across our treatment groups. Furthermore, similar to *Bdnf, Lept-R, Oxy-R* and *Tert*, *Igf2-R* methylation was influenced by paternal experience, providing further support that manipulation of paternal characteristics can modulate developmental programming of offspring^12, 72^.

Finally, given that evidence suggests TL is inherited in a father-to-offspring manner,^73^, and changes in TL have been linked to environmental factors^39, 41, 74–77^, this study also examined transmission of TL from fathers to their sons, as it pertained to the three distinct paternal groups. Consistent with previous studies, TL in AA father’s sperm was significantly longer than TL in the control or HFD fathers. Although most cells undergo aging-dependent reductions in TL, sperm have been identified as one of the only tissues that exhibit TL elongation^78, 79^. In both the HFD and Control groups, TL was consistent across fathers and their sons, and for sons there were no differences in TL across brain and sperm samples. However, sons from the AA group exhibited shorter sperm TL than their fathers, but this significant decrease was not present in the brain; brain TL did not differ from TL in father’s sperm. It may be possible that differences in TL take time to manifest, and the young age of sacrifice for offspring in this study (P60) did not allow for the emergence of these changes. It is however interesting to note that our previous studies have demonstrated mTBI-induced reductions in brain and skin TL^42, 80^, but this was not identified in sperm.

In summary, increasing evidence implicates epigenetic change in sperm as the primary mechanism by which paternal effects are transmitted across generations^81, 82^. This study provides further support for the epigenetic transmission of traits from fathers to sons by demonstrating that DNA methylation of specific promoter regions for certain genes is highly conserved across generations. Although only speculative, the changes identified in this study are likely quite stable. Given that epigenomes are highly plastic during development, but ultimately form methylation patterns that are tightly regulated and surprisingly non-random in adulthood^83, 84^, paternal effects identified in adolescent offspring would be considered stable modifications. As the range of factors that contribute to an individual’s phenotype is vast and can be described as an amalgamation of traits that arise from genetic, epigenetic, and environmental influences^12^, it is not possible to ascertain the magnitude of influence these paternal experiences have on individual heterogeneity. However, future studies must acknowledge that paternal experience does influence offspring brain and behavior and may modify outcomes.

Many epidemiological studies have demonstrated that paternal characteristics (age and diet specifically) do influence offspring well being^18–20, 23^. Therefore, although we examined outcomes in a highly controlled animal study, there is evidence to support paternal transmission of risk within the complexities of human life. In this study, we found that while contributing to offspring fitness and phenotype, paternal experiences also mediated individual differences in susceptibility to post-injury symptomology by modifying DNA methylation of specific genes. Interestingly, a single concussion was also able to change the promoter DNA methylation pattern in 3/6 of our genes of interest, suggesting that a history of mTBI may influence gene expression in subsequent generations. This notion will require further investigation and multigenerational studies, but has significant implications for many people in groups at high risk for mTBI, especially military personnel and professional athletes.

## Methods

This study was built upon results from a previously published study that was conducted in our laboratory^24^. All experiments were conducted in accordance with the Canadian Council on Animal Research and were approved by the University of Calgary Conjoint Faculties Research Ethics Board. Briefly, in the prior study, twelve male rats were assigned to one of three groups (Advanced Age (AA), High Fat Diet consumption (HFD), or Control) and were mated with naïve females (all females were 65 days of age at the time of mating). Pups were permitted to develop without further intervention and experienced a mTBI or a sham injury at postnatal day 30 (P30), which was followed by a comprehensive behavioural test battery. The study demonstrated that paternal experience prior to conception altered both premorbid characteristics of offspring and their pathophysiological response to the mTBI^24^.

The main purpose of the current study was to explore epigenetic modes of inheritance involved in the transmission of paternal experiences to differential outcomes in offspring. The current study utilized sperm and brain tissue from the animals when they were sacrificed. Fathers were sacrificed immediately after mating (P65 – HFD and Control, P105 – AA) and sperm samples were collected. Once behavioural testing was complete, pups were sacrificed, sperm samples, and tissue from the hippocampus (HPC) of the brain was collected from all male pups at P60. We used the novel MethyLight technique to determine methylation levels of promoter regions for specific genes in our samples. MethyLight distinguishes between methylated and unmethylated CpG islands in the gene’s promoter region by combining methylation specific priming with probe binding fluorescence; hence fluorescence is observed when a probe binds to a methylated promoter region. In order to determine if changes in DNA methylation actually affected gene expression, we compared gene methylation and gene expression changes in the offspring brains.

### DNA and RNA Extraction

The rats were anesthetized with isofluorane until the toe-pinch hind leg reflex was not observed, and were quickly decapitated. The epididymis was isolated from male rats, punctured with small holes using a needle, and incubated at 37 °C in 350 µL of phosphate buffered saline (PBS) for 30 minutes, to permit swim out of the sperm. The supernatant was transferred to a new tube and additional 350 µL PBS was added to the tube containing the epididymis. The new tube was allowed to incubate at 37 °C for an additional 30 minutes and the supernatant was removed again. The two supernatants were pooled and centrifuged at 4 °C. A pellet was obtained and sperm DNA was extracted using the Qiagen DNA Micro kit (Qiagen, Germany). At the time of sacrifice for male pups, the brain was quickly removed, weighed, and the HPC was extracted and flash frozen on dry ice. DNA and RNA were extracted from the HPC using the RNA/DNA Mini kit according to manufacturer’s protocols (Qiagen, Germany).

### Bisulfite Conversion and MethyLight Reactions

Genomic DNA from paternal and male offspring sperm, as well as the HPC, was bisulfite converted using Qiagen’s EpiTect Fast DNA Bisulfite (Conversion) Kit according to the manufacturer’s protocol prior to methylation analysis. In order to examine specific methylation patterns the MethyLight technique was utilized. To investigate inheritance mechanisms, methylation patterns of the promoter regions in the same genes were examined in the father and offspring sperm, and offspring brain. Primers specific for regions high in CpG islands in the promoter of each of our target genes were designed in house using MethPrimer^85^. Each gene’s promoter region was determined from a rat promoter database (https://cb.utdallas.edu/cgi-bin/TRED/tred.cgi?process=searchPromForm). If the promoter did not exist in this database, we used Ensembl’s gene database (http://www.ensembl.org/index.html) and input it into the promoter prediction software (http://www.cbs.dtu.dk/services/Promoter/). The entire promoter region was entered into Methprimer and Methprimer generated the CpG island(s) from which we chose our primers and probes. See Table 6. The Qiagen EpiTect Methylight PCR kit, following the manufacturer’s protocol “Methylation-specific real-time PCR analysis using TaqMan probes (other instruments)” was used. A 96 well plate was used and each reaction was completed in duplicate using 10 ng of DNA. Additionally, no template controls (NTCs) were loaded on all plates to monitor reaction quality. FAM (methylated probe fluorescence) and VIC (unmethylated probe fluorescence) were measured in each of the wells. For analysis, test samples were compared to reference DNA with known methylation (100% and 0% methylated). Methylation levels were computed in a manner similar to that described elsewhere^86^ (% methylation = 100/[1 + 2^**^**(CtFAM – CtVIC)^]).

**Table 6:** Methylight RT-qPCR primers and methylation-specific probes.

	***Bdnf***
**Forward Primer**	5′-TTTGGTGTAGAAATATTTGGAGTTAG-3′
**Reverse Primer**	5′-CAAAAAAAATCACAATAAAATACCC-3′
**Probes (Methylated)**	5′-/6FAM/GCGCGTATATATATATACGG/MGBNFQ/-3′
	5′-/6FAM/GCGTGTATATATATATACGG/MGBNFQ/-3′
	5′-/6FAM/GTGCGTATATATATATATGG/MGBNFQ/-3′
**Probe (Unmethylated)**	5′-/VIC/GTGTGTATATATATATATGG/MGBNFQ/-3′
	***Lept-R***
**Forward Primer**	5′-GGTTGAGATTAGAAGTGGAGATTT-3′
**Reverse Primer**	5′-ATCCTACCTCCAACCCATAAAAA-3′
**Probes (Methylated)**	5′-/6FAM/TCGGGGGCGGAGTTTCGAAG/MGBNFQ/-3′
	5′-/6FAM/TCGGGGGTGGAGTTTCGAAG/MGBNFQ/-3′
	5′-/6FAM/TTGGGGGCGGAGTTTTGAAG/MGBNFQ/-3′
**Probe (Unmethylated)**	5′-/VIC/TTGGGGGTGGAGTTTTGAAG/MGBNFQ/-3′
	***Oxy-R***
**Forward Primer**	5′-TGTTAGGAGGAGAATTATTTGTTT-3′
**Reverse Primer**	5′-CTACTAAACCCCCTCCTACTCAC-3′
**Probes (Methylated)**	5′-/6FAM/GCGTTATCGCGGGTATTT/MGBNFQ/-3′
	5′-/6FAM/GCGTTATTGCGGGTATTT/MGBNFQ/-3′
	5′-/6FAM/GTGTTATCGTGGGTATTT/MGBNFQ/-3′
**Probe (Unmethylated)**	5′-/VIC/GTGTTATTGTGGGTATTT/MGBNFQ/-3′
	***Tert***
**Forward Primer**	5′-TTGGTTAGTAGTTTTAAGTATTAGTTGTGG-3′
**Reverse Primer**	5′-TCAAAATCCCAACCATTAAAAAC-3′
**Probes (Methylated)**	5′-/6FAM/GCGTTTTCGTTTTTTTCGTT/MGBNFQ/-3′
	5′-/6FAM/GCGTTTTTGTTTTTTTCGTT/MGBNFQ/-3′
	5′-/6FAM/GTGTTTTCGTTTTTTTTGTT/MGBNFQ/-3′
**Probe (Unmethylated)**	5′-/VIC/GTGTTTTTGTTTTTTTTGTT/MGBNFQ/-3′
	***Igf2***
**Forward Primer**	5′-TTTTTTATATTGTTTAAATAAAATTGTTTT-3′
**Reverse Primer**	5′-ACCCCCAATAATTACCCCTAAC-3′
**Probes (Methylated)**	5′-/6FAM/CGTTAGGGAGCGTATAAGGTCGGAT/MGBNFQ/-3′
	5′-/6FAM/CGTTAGGGAGTGTATAAGGTCGGAT/MGBNFQ/-3′
	5′-/6FAM/TGTTAGGGAGCGTATAAGGTTGGAT/MGBNFQ/-3′
**Probe (Unmethylated)**	5′-/VIC/TGTTAGGGAGTGTATAAGGTTGGAT/MGBNFQ/-3′
	***Igf-R***
**Forward Primer**	5′-TGGGTTTTTTTATTTAATTTTATTT-3′
**Reverse Primer**	5′-CAAAACCCAAACCTCAATTTC-3′
**Probes (Methylated)**	5′-/6FAM/GCGTTTAAGCGTAGACGTAA/MGBNFQ/-3′
	5′-/6FAM/GCGTTTAAGTGTAGACGTAA/MGBNFQ/-3′
	5′-/6FAM/GTGTTTAAGCGTAGATGTAA/MGBNFQ/-3′
**Probe (Unmethylated)**	5′-/VIC/GTGTTTAAGTGTAGATGTAA/MGBNFQ/-3′

### RT-qPCR

Quantitative real time PCR (RT-qPCR) was performed on cDNA synthesized from the pup’s brain tissue to determine if changes in promoter methylation are correlated with changes in gene expression. Primer sequences and cycling parameters can be found in detail in prior studies carried out in this laboratory^80^. The 2^−ΔΔCt^ method, as described by Pfaffl^87^, normalized against two housekeeping genes (*Ywhaz* and *CycA*) was used^88^. Ten nanograms of cDNA with 0.5 μM of each of the forward and reverse primers and 1x SYBR Green FastMix with Rox was used for RT-qPCR analysis on the CFX Connect-Real-Time PCR Detection system (BioRad, Hercules, CA). Each sample was tested in duplicate and PCR efficiency was between 91.5 and 105.8%.

### Telomere Length Analysis

Telomere Length (TL) analysis was conducted on all DNA samples using a protocol similar to that described by Cawthon^89^ and used in Hehar and Mychasiuk^42^. Genomic DNA samples were diluted to a concentration of 10 ng/μl. PCR reactions were conducted by adding 1 µL DNA, 1 × SYBR Green FastMix with Rox for RT-qPCR, and primers so that the total volume in each well was 20 µL. Each reaction was performed in duplicates and NTCs were also loaded onto the 96 well plate to ensure that the reactions were not contaminated. The final concentrations for primers were: 270 nM for Tel forward; 900 nM for Tel reverse; 300 nM for 36B4 forward, and 500 nM for 36B4 reverse. Absolute quantitative PCR was used to determine the ratio of telomeres to a single copy gene (*36B4*). Thus, two reaction plates were conducted, one for TL and another for *36B4* and a ratio of TL to *36B4* was determined. When the ratio of telomere to single copy gene (T/S) is 1, it suggests that the sample DNA has an identical telomere repeat and single copy number as the target DNA. The T/S ratio was calculated to be approximately [2^C^t^(telomeres)^/2^C^t^(36B4)^]^−1^ = −2^−∆^^C^t. This ratio was then used in the linear regression equation described by Cawthon^89^, y = 1910.5x + 4157, in which y = TL and x = −2^−∆^^C^t, to determine the TL of each sample.

### Statistical Analysis

All statistical analyses were carried out with SPSS 22.0 for Mac. One-way ANOVAs were performed for each of the molecular measures examined. The factors used in the various ANOVAs were Paternal Treatment (AA, HFD, Control), Generation (Fathers, Sons), Tissue Sampled (Sperm, Brain), and Injury (mTBI, Sham). Pearson’s correlations between methylation level and expression were run for each of the genes for the given paternal treatment groups. *p* < 0.05 was considered statistically significant and Tukey’s pairwise post-hoc analyses were conducted where appropriate.
